# Spin-orbit coupling effects on transport properties of electronic Lieb lattice in the presence of magnetic field

**DOI:** 10.1038/s41598-022-12588-5

**Published:** 2022-05-20

**Authors:** Elham Sadeghi, Hamed Rezania

**Affiliations:** Physics Department, Razi Univerity, Kermanshah, Iran

**Keywords:** Electronic properties and materials, Physics

## Abstract

In this paper, the transport properties of a two-dimensional Lieb lattice that is a line-centered square lattice are investigated in the presence of magnetic field and spin-orbit coupling. Specially, we address the temperature dependence of electrical and thermal conductivities as well as Seebeck coefficient due to spin-orbit interaction. We have exploited Green’s function approach in order to study thermoelectric and transport properties of Lieb lattice in the context of Kane–Mele model Hamiltonian. The results for Seebeck coefficient show the sign of thermopower is positive in the presence of spin-orbit coupling. Also the temperature dependence of transport properties indicates that the increase of spin-orbit coupling leads to decrease thermal conductivity however the decrease of gap parameter causes the reduction of thermal conductivity. There is a peak in temperature dependence of thermal conductivity for all values of magnetic fields and spin-orbit coupling strengths. Both electrical and thermal conductivities increase with increasing the temperature at low amounts of temperature due to the increasing of transition rate of charge carriers and excitation of them to the conduction bands. Also we have studied the temperature dependence of Seebeck coefficient of Lieb monolayer due to both spin orbit coupling and magnetic field factors in details.

## Introduction

A line-centered square lattice, called Lieb lattice^1^ has attracted much attention both theoretically and experimentally as its novel topological properties. This lattice includes three atoms in a square unit cell. Moreover the energy spectrum of this structure is characterized by a three band structure with particle-hole symmetry and a flat band touching two linearity dispersing interesting bands at a Dirac point. It is well established that Dirac cones cause to unusual behavior, such as effectively massless fermions. Flat bands may potentially facilitate the realization of magnetic order^2^ causes to the fractional Hall effect^3,4^ and increase the superconducting transition temperature^5^. The Lieb lattice introduces an interesting lattice structure for magnetism topic so that the theoretical studies on the flat-band ferromagnetism in the two dimensional Lieb lattice have been performed^6,7^. In the Cu$O_{2}$ planes of cuprate superconductors, atoms are just arranged in the Lieb lattice^8,9^. Moreover the Lieb lattice can be realized in optical lattices^3^.

One of the most important models for describing topological insulator as quantum spin Hall effect with a low-energy Dirac structure is the 2 dimensional Lieb lattice. Such model have been shown to support topological states^10,11^. The original model on the Lieb lattice was realized by superposing two copies of the Haldane model^12^, one for spin up and other for spin down electrons moving in opposite directions along the edge^13^. The experimental results show the realization of a dispersionless localized flat-band state in Lieb photonic lattices^14^.

The intrinsic spin-orbit coupling is applied to the Lieb lattice so that a topologic bulk gap is opened and it gives rise to the quantum spin Hall effect characterized by two pairs of gapless helical edge states within the bulk gap^15^. The topological phase transition due to different parameters on the Lieb lattice have also been further addressed^16–18^. Moreover, it was also found that the edge geometries of two-dimensional Lieb lattice topological insulators have significant influences on the edge modes and the finite size effect of quantum spin Hall states^19,20^.

It was found that intrinsic spin-orbit coupling due to a perpendicular electric field or interaction with a substrate plays an important role on the topological properties of nano structures. It was predicted that spin orbit coupling and exchange field together open a nontrivial bulk gap in two dimensional non structures leading to the quantum spin hall effect^21,22^. The topological phase transitions in the 2D crystals can be understood based on intrinsic spin orbit coupling which arises due to perpendicular electric field or interaction with a substrate.

A simple model introduced by Kane and Mele^23^ has been applied to describe topological insulators. Such model consists of a hopping and an intrinsic spin-orbit term on the nano structures. The Kane–Mele model essentially includes two copies with different sign for up and down spins of a model introduced earlier by Haldane^12^. Moreover it has been shown that in-plane magnetic field induces two dimensional Lieb lattice magneto resistance which is negative for extrinsic gapless structure. Also magneto-resistance has a positive value for fields lower than the critical magnetic field and negative above the critical magnetic^24^. Microwave magneto transport in doped Lieb lattice is an open problem^25^.

The well prepared samples of nanostructures such as Lieb lattice have high electric and thermal conductivity, high transparency with respect to white light, impermeability to gases, and the ability to be chemically functionlized^26^. Electronic gaps in Lieb lattice can be controlled and thus this nanostructure is an extremely flexible. This can be accomplished with an electric or magnetic field applied perpendicular to the plane. It was shown theoretically^27,28^ and demonstrated experimentally^29,30^ that a Lieb nanostructure is the only material with semiconducting properties that can be controlled by electric field effect^31^. The size of the gap between conduction and valence bands depending on spin orbit coupling strength and various atoms on different sublattices can be as large as 0.1–0.3 eV, allowing for novel terahertz devices^30^.

Static transport in some of the nanostructures have been studied for both without and in the presence of a magnetic field. It is established that charged impurity scattering is primarily responsible for the transport behavior observed in monolayer graphene^32,33^. A comprehensive study of the electronic properties of the carbon based nanostructure in the presence of defects as a function of temperature, external frequency, gate voltage, and magnetic field has been presented by Peres and coworkers^34^. Thus the investigation of transport properties of low dimensional Lieb lattice in the presence of spin-orbit coupling and magnetic field can be an interesting theoretical topic in nanostructures studies.

Full band calculation has been implemented to derive in-plane transport properties of the structure. We have exploited Green’s function approach^35^ to calculate the transport coefficients, i.e. the time ordered heat and electrical current correlation functions. Using transport coefficients, we can find the electrical and thermal conductivities. Moreover the sign of thermopower (Seebeck coefficient) determines the sign of majority of charge carriers. A negative (positive) thermopower shows that negative (positive) charge carriers, i.e. electrons (holes), in transport process are dominant. The effects of coupling of electron spin degree of freedom with magnetic field on the transport and thermoelectric properties of Lieb nanostructure have been studied via adding the Zeeman term to the original model Hamiltonian. Also we discuss and analyze to show how spin-orbit coupling strength and magnetic field affects the temperature dependence of the electrical and thermal conductivities and Seebeck coefficient of Lieb nano lattice.

The aim of this paper is to provide a Kane Mele model including intrinsic spin-orbit interaction for studying the transport properties of monolayer Lieb lattice in the presence of magnetic field perpendicular to the plane. Using the spin-orbit coupling strength and on-site energy parameter values, the band dispersion of electrons on Lieb lattice has been calculated. The difference between on-site energies of sublattice atoms is introduced by gap parameter. We have exploited Green’s function approach to calculate the transport coefficients, i.e. the time ordered correlation between heat and electrical currents. Specially we have calculated electrical conductivity, thermal conductivity and Seebeck coefficient as transport properties of Lieb lattice. The effects of coupling of electron spin degree of freedom with magnetic field on the conductivities and Seebeck coefficient have been studied via adding the Zeeman term to the original model Hamiltonian. Also we discuss and analyze to show how gap parameter affects the temperature dependence of the thermal and electrical conductivities. Also we study the behavior of static thermal conductivity and Seebeck coefficient of Lieb lattice versus temperature due to the magnetic field effects. Moreover the effects of spin-orbit coupling on transport and thermoelectric properties of Lieb lattice have been addressed in details.

## Model Hamiltonian and density of states

**Figure 1:**
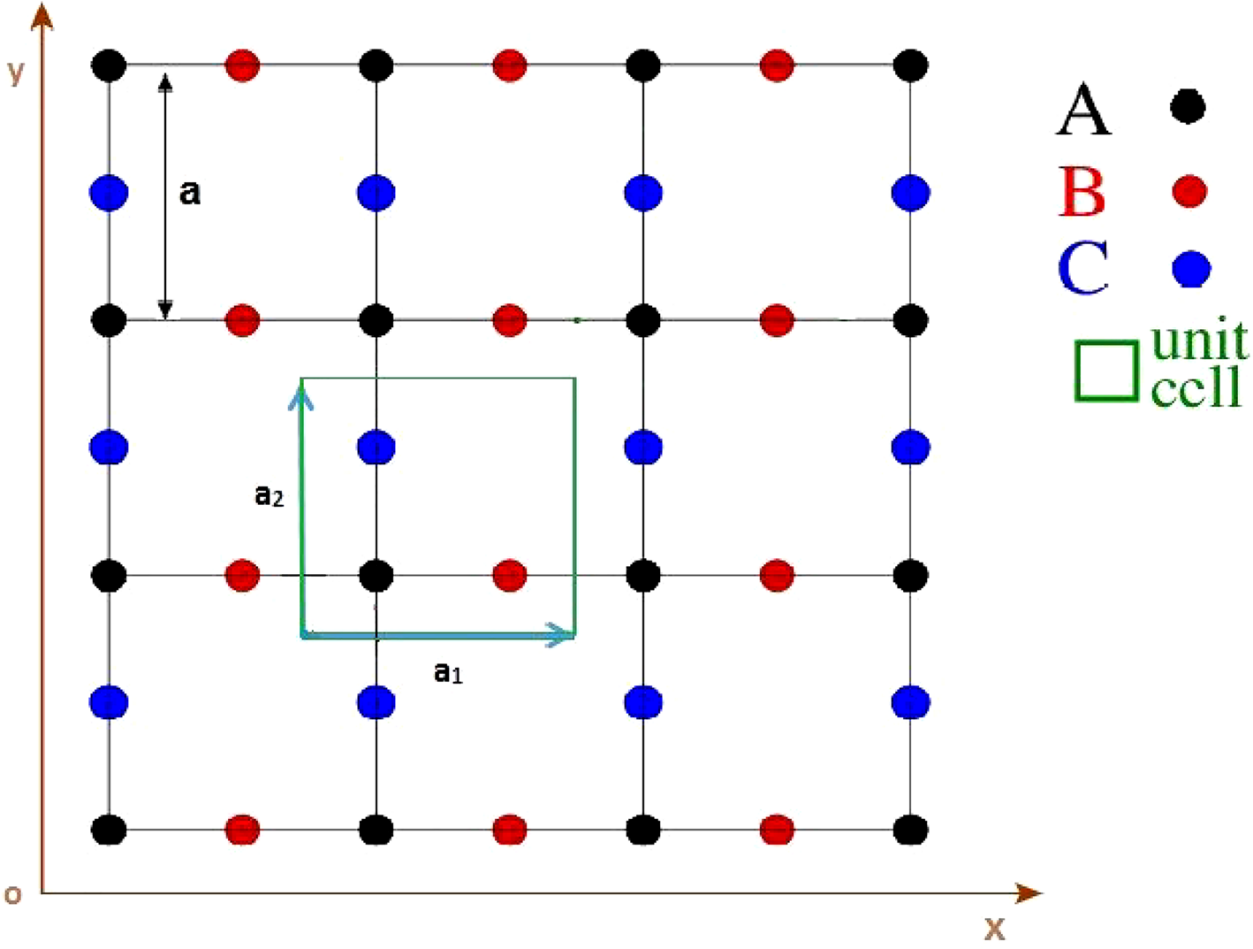
Lieb lattice showing three-site basis in unit cell, with unit cell vectors $\mathbf{a}_{1},\mathbf{a}_{2}$.

The crystal structure of Lieb lattice has been shown in left panel of Fig. 1. Each unit cell on the Lieb lattice contains three atoms (A, B and C) according to left panel of Fig. 1. The primitive unit cell vectors of Lieb lattice have been shown by $\mathbf{a}_{1}=a\mathbf{i}$ and $\mathbf{a}_{2}=a\mathbf{j}$ so that $\mathbf{i}$ and $\mathbf{j}$ denote the unit vectors along the *x* and *y* axis respectively. Also *a* implies the length of each unit cell vector. We will assume only single electron orbital gives the major contribution to the band structure of electrons.

In the presence of longitudinal magnetic field, the Kane–Mele model^23^ (*H*) for Germanene structure includes the tight binding model ($H^{TB}$), the intrinsic spin-orbit coupling ($H^{ISOC}$) and the Zeeman term ($H^{Zeeman}$) due to the coupling of spin degrees of freedom of electrons with external longitudinal magnetic field *B*

1 $$\begin{aligned} H= &  H^{TB}+H^{ISOC}+H^{Zeeman}. \end{aligned}$$

The tight binding part of model Hamiltonian consists the nearest neighbor hopping parameter *t*. The tight binding part of model Hamiltonian under nearest neighbor approximation takes the following form

2 $$\begin{aligned} H^{TB}= &  -t\sum _{\langle iA,jB\rangle ,\sigma }\Big (c^{\sigma \dag }_{j,B}c^{\sigma }_{i,A}+h.c.\Big ) -t\sum _{\langle iA,jC\rangle ,\sigma }\Big (c^{\sigma \dag }_{j,C}c^{\sigma }_{i,A}+h.c.\Big )\nonumber \\ + &  \Delta \sum _{i,\sigma }c^{\sigma \dag }_{i,B}c^{\sigma }_{i,B}-\Delta \sum _{i,\sigma }c^{\sigma \dag }_{i,C}c^{\sigma }_{i,C} -\mu \sum _{i,\sigma }\sum _{\alpha =A,B,C} c^{\sigma \dag }_{i,\alpha }c^{\sigma }_{i,\alpha }. \end{aligned}$$

Also the intrinsic spin-orbit coupling and Zeeman terms of model Hamiltonian are given by^23^

3 $$\begin{aligned} H^{ISOC}= &  i\lambda \sum _{\langle \langle i\alpha ,j\beta \rangle \rangle ,\sigma } \Big ((\mathbf{d}_{j\beta }\times \mathbf{d}_{i\alpha })\cdot \tilde{\sigma}_{\sigma \sigma '}c^{\dag \sigma }_{j,\beta } c^{\sigma '}_{i,\alpha }\Big ),\nonumber \\ H^{Zeeman}= &  -\sum _{i,\sigma ,\alpha }\sigma g\mu _{B}B\Big (c^{\sigma \dag }_{i,\alpha }c^{\sigma }_{i,\alpha }\Big ). \end{aligned}$$

Here $c^{\sigma }_{i,\alpha }$ is an annihilation operator of electron with spin $\sigma$ on sublattice $\alpha =A,B,C$ in unit cell with index *i*. The operators fulfill the fermionic standard anti commutation relations $\{c^{\sigma }_{i,\alpha },c^{\sigma '\dag }_{j,\beta }\}= \delta _{ij}\delta _{\sigma \sigma '}\delta _{\alpha ,\beta }$^35^. As usual *t* denotes the nearest neighbor hopping integral amplitude. We consider the sublattice symmetry breaking mechanism in which the on-site energies for A, B and C sublattices are different.

**Figure 2:**
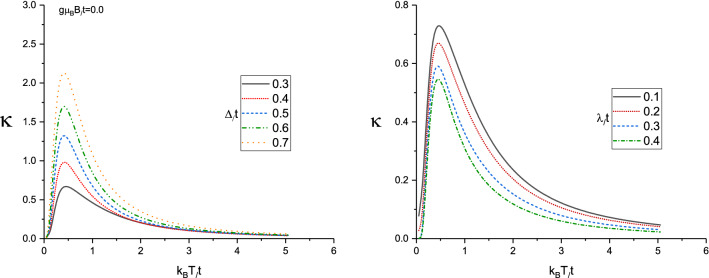
Left Panel: Thermal conductivity of Lieb lattice as a function of normalized temperature $k_{B}T/t$ for different values of normalized gap parameter $\Delta /t$ in the absence of magnetic field. Normalized chemical potential and normalized spin-orbit coupling have been fixed at $\mu /t=1.0$ and $\lambda /t=0.2$, respectively. Right Panel: Thermal conductivity of Lieb lattice as a function of normalized temperature $k_{B}T/t$ for different values of normalized spin-orbit coupling $\lambda /t$ in the absence of magnetic field. Normalized chemical potential and normalized gap parameter have been fixed at $\mu /t=1.0$ and $\Delta /t=0.3$, respectively.

Based on Eq. (2), these different on-site energies are named $\Delta$ and $-\Delta$ for B and C sublattices, respectively. It should be noted that the on site energy for sublattice *A* is considered to be zero. The parameter $\lambda$ introduces the spin-orbit coupling strength. Since $\lambda$ describes the spin-orbit coupling strength and *t* introduces the hopping amplitude of electrons and *t* is related to kintetic energy of electrons, there is no relation between hopping amplitude *t* and $\lambda$. In other words spin orbit coupling has no effect on *t*. Also *B* refers to strength of applied magnetic field. *g* and $\mu _{B}$ introduce the gyromagnetic and Bohr magneton constants, respectively. $\vec{\sigma }$ is the vector of Pauli spin matrices. Based on Fig. 2, $\mathbf{a}_{1}$ and $\mathbf{a}_{2}$ are the primitive vectors of unit cell and the length of them is assumed to be unit.

We consider the intrinsic spin-orbit term^23^ of the KM Hamiltonian in Eq. (2). Since the operator form of $H^{ISOC}$ in Eq. (3) makes the coupling between next nearest neighbor lattice sites and based on crystal structure of Lieb lattice in left panel of Fig. 1, the sublattices indices $\alpha ,\beta$ in Eq. (3) should be $\alpha =B, \beta =C$ or $\alpha =C,\beta =B$. In addition, $\mathbf{d}_{j\beta }$ and $\mathbf{d}_{i\alpha }$ in Eq. (3) are the two unit vectors along the nearest neighbor bonds connecting lattice site in unit cell *i* on sublattice $\alpha$ to its next-nearest neighbor site in unit cell *j* on sublattice $\beta$.

Because of three sublattice atoms, the band wave function $\psi _{n}(\mathbf{k},\mathbf{r})$ can be expanded in terms of Bloch functions $\Phi _{\alpha }(\mathbf{k},\mathbf{r})$. The index $\alpha$ implies two inequivalent sublattice atoms *A*, *B*, *C* in the unit cell, $\mathbf{r}$ denotes the position vector of electron, $\mathbf{k}$ is the wave function belonging in the first Brillouin zone of square structure, i,e. $-\frac{\pi }{2a}<k_{x}<\frac{\pi }{2a}$, $-\frac{\pi }{2a}<k_{y}<\frac{\pi }{2a}$ . Such band wave function can be written as^36^

4 $$\begin{aligned} \psi _{n}(\mathbf{k},\mathbf{r})=\sum _{\alpha =A,B,C}C^{n}_{\alpha }(\mathbf{k}) \Phi _{\alpha }(\mathbf{k},\mathbf{r}), \end{aligned}$$

where $C^{n}_{\alpha }(\mathbf{k})$ is the expansion coefficients and *n* refers to energy band spectrum index. Also we expand the Bloch wave function in terms of Wannier wave function as^36^

5 $$\begin{aligned} \Phi _{\alpha }(\mathbf{k},\mathbf{r})=\frac{1}{\sqrt{N}}\sum _{\mathbf{R}_{i}} e^{i\mathbf{k}.\mathbf{R}_{i}}\phi _{\alpha }(\mathbf{r}-\mathbf{R}_{i}), \end{aligned}$$

so that $$\mathbf{R}_{i}$$ implies the position vector of *i*th unit cell in the crystal and $$\phi _{\alpha }$$ is the Wannier wave function of electron in the vicinity of atom in *i* th unit cell on sublattice index $$\alpha $$. The matrix forms of $$H^{TB}$$ and $$H^{ISOC} $$in Bloch wave function space **are** introduced by

6 $$\begin{aligned} {{\mathcal {H}}}^{TB}_{\sigma }(\mathbf{k})= &  \left( \begin{array}{ccc} H^{TB}_{AA}(\mathbf{k})&\quad H^{TB}_{AB}(\mathbf{k})&\quad H^{TB}_{AC}(\mathbf{k}) \\ H^{TB}_{BA}(\mathbf{k}) &\quad H^{TB}_{BB}(\mathbf{k})&\quad H^{TB}_{BC}(\mathbf{k}) \\ H^{TB}_{AA}(\mathbf{k})&\quad H^{TB}_{AB}(\mathbf{k})&\quad H^{TB}_{CC}(\mathbf{k})\\ \end{array} \right) ,\nonumber \\ {{\mathcal {H}}}^{ISOC}_{\sigma }(\mathbf{k})= &\quad  \left( \begin{array}{ccc} H^{ISOC}_{AA}(\mathbf{k})&\quad H^{ISOC}_{AB}(\mathbf{k})&\quad H^{ISOC}_{AC}(\mathbf{k}) \\ H^{ISOC}_{BA}(\mathbf{k}) &\quad H^{ISOC}_{BB}(\mathbf{k})&\quad H^{ISOC}_{BC}(\mathbf{k}) \\ H^{ISOC}_{AA}(\mathbf{k})&\quad H^{ISOC}_{AB}(\mathbf{k})&\quad H^{ISOC}_{CC}(\mathbf{k})\\ \end{array} \right) . \end{aligned}$$

A note is in order here. Based on commutation of total model Hamiltonian *H* with Pauli matrix $\sigma _{z}$, the matrix representation of Hamiltonian in Eqs. (1, 2 and 3) is diagonal in Hilbert space of eigenfunctions of Pauli matrix $\sigma _{z}$ and moreover the matrix elements of ${{\mathcal {H}}}^{TB}$ and ${{\mathcal {H}}}^{ISOC}$ are independent of spin index quantum number $\sigma =\uparrow ,\downarrow$. Using the Bloch wave functions, i.e. $\Phi _{\alpha }(\mathbf{k})$, the matrix elements of ${{\mathcal {H}}}$ are given by

7 $$\begin{aligned} H^{TB}_{\alpha \beta }(\mathbf{k})=\langle \Phi _{\alpha }(\mathbf{k})| H^{TB}|\Phi _{\beta }(\mathbf{k})\rangle \;\;,\;\; H^{ISOC}_{\alpha \beta }(\mathbf{k})=\langle \Phi _{\alpha }(\mathbf{k})| H^{ISOC}|\Phi _{\beta }(\mathbf{k})\rangle . \end{aligned}$$

Up to nearest neighbor approximation, one can obtain the matrix form of tight binding part of total Hamiltonian, i.e. ${\mathcal H}^{TB}$, as follows

8 $$\begin{aligned} {{\mathcal {H}}}^{TB}_{\sigma }(\mathbf{k})=\left( \begin{array}{ccc} -\mu & 2tcos(k_{x}a/2)&2tcos(k_{y}a/2) \\ 2tcos(k_{x}a/2) & \Delta -\mu &0 \\ 2tcos(k_{y}a/2)& 0&-\Delta -\mu \\ \end{array} \right) . \end{aligned}$$

The based vectors connecting next nearest neighbor atomic sites in left panel of Fig. 1 are given by

9 $$\begin{aligned} \mathbf{R}_{1}=-\frac{a}{2} \mathbf{i}-\frac{a}{2} \mathbf{j}\;\;,\;\;\mathbf{R}_{2}=\frac{a}{2} \mathbf{i}-\frac{a}{2} \mathbf{j},\nonumber \\ \mathbf{R}_{3}=\frac{a}{2} \mathbf{i}+\frac{a}{2} \mathbf{j}\;\;,\;\;\mathbf{R}_{4}=-\frac{a}{2} \mathbf{i}+\frac{a}{2} \mathbf{j}, \end{aligned}$$

so that the matrix form of ${{\mathcal {H}}}^{ISOC}$ is obtained by

10 $$\begin{aligned} {{\mathcal {H}}}^{ISOC}_{\sigma }(\mathbf{k})=\left( \begin{array}{ccc} 0& 0&0\\ 0 & 0&-4i\lambda sin(k_{x}a/2)sin(k_{y}a/2)\\ 0& 4i\lambda sin(k_{x}a/2)sin(k_{y}a/2)&0\\ \end{array} \right) . \end{aligned}$$

Using the matrix forms in Eqs. (8 and 10), the total model Hamiltonian in Eq. (1) takes the following form

11 $$\begin{aligned} {{\mathcal {H}}}_{\sigma }(\mathbf{k})=\left( \begin{array}{ccc} -\mu -\sigma g\mu _{B}B& 2tcos(k_{x}a/2)&2tcos(k_{y}a/2) \\ 2tcos(k_{x}a/2) & \Delta -\mu -\sigma g\mu _{B}B&-4i\lambda sin(k_{x}a/2)sin(k_{y}a/2) \\ 2tcos(k_{y}a/2)& 4i\lambda sin(k_{x}a/2)sin(k_{y}a/2)&-\Delta -\mu -\sigma g\mu _{B}B\\ \end{array} \right) . \end{aligned}$$

The matrix elements of ${{\mathcal {H}}}_{\sigma }(\mathbf{k})$ are expressed based on hopping amplitude and spin-orbit coupling between two neighbor atoms on lattice sites and can be expanded in terms of hopping amplitude *t*, spin orbit coupling $\lambda$ and gap parameter $\Delta$. The off-diagonal elements of matrixes ${\mathcal H}$ in Eq. (11) arise from hopping amplitude of electrons between nearest neighbor atoms on the different sublattices and spin-orbit coupling. Using the Hamiltonian form in Eq. (11), the band structure of electrons, i.e. $E^{\sigma }_{\eta }(\mathbf{k})$ has been found by solving equation $\det \Big ({{\mathcal {H}}}(\mathbf{k})-E(\mathbf{k})\Big )=0$. We have applied the following definitions

12 $$\begin{aligned} \chi _{x}\equiv 2tcos(k_{x}a/2)\;\;,\;\;\chi _{y}\equiv 2tcos(k_{y}a/2)\;\;,\;\;\chi \equiv 4\lambda sin(k_{x}a/2)sin(k_{y}a/2). \end{aligned}$$

In Appendix A, we have presented the explicit relations of electronic band structure of total Hamiltonian in Eq. (11). Using band energy spectrum, the Hamiltonian of Lieb lattice in Eq. (1) can be rewritten by^36^

13 $$\begin{aligned} H=\sum _{\mathbf{k},\sigma ,\eta =1,2,3} E^{\sigma }_{\eta }(\mathbf{k})c^{\dag \sigma }_{\eta ,\mathbf{k}}c^{\sigma }_{\eta ,\mathbf{k}}. \end{aligned}$$

where $c^{\sigma }_{\eta ,\mathbf{k}}$ defines the creation operator of electron with spin $\sigma$ in band index $\eta$ at wave vector $\mathbf{k}$. The electronic Green’s function can be defined using the Hamiltonian in Eq. (13) as following expression^35^

14 $$\begin{aligned} G^{\sigma }_{\eta }(\mathbf{k},\tau )=-\langle T_{\tau } c^{\sigma }_{\eta ,\mathbf{k}}(\tau )c^{\dag \sigma }_{\eta ,\mathbf{k}}(0)\rangle , \end{aligned}$$

where $\tau$ is imaginary time. Using the model Hamiltonian in Eq. (13), the Fourier transformations of Green’s function is given by^35^

15 $$\begin{aligned} G^{\sigma }_{\eta }(\mathbf{k},i\omega _{n})=\int ^{1/k_{B}T}_{0}d\tau e^{i\omega _{n}\tau } G^{\sigma }_{\eta }(\mathbf{k},\tau )=\frac{1}{i\omega _{n}-E^{\sigma }_{\eta }(\mathbf{k})}. \end{aligned}$$

Here $\omega _{n}=(2n+1)\pi k_{B}T$ denotes the fermionic Matsubara frequency^35^ in which *T* is equilibrium temperature. The electronic density of states of Lieb structure in the presence of intrinsic spin-orbit and external magnetic field can be obtained by electronic band structure as

16 $$\begin{aligned} D(E)=-\frac{1}{6\pi N}\sum _{\mathbf{k},\sigma }\sum _{\eta =1}^{3}\mathrm Im G^{\sigma }_{\eta \eta }(\mathbf{k},E)=-\frac{1}{6N}\mathrm{Im}\sum _{\mathbf{k}}\sum _{\eta =1}^{3} \frac{1}{E-E^{\sigma }_{\eta }(\mathbf{k})+i0^{+}}, \end{aligned}$$

Summation over wave vectors have been performed into first Brillouin zone of Lieb lattice. The density of states includes prominent asymmetric peaks due to the band edge of parabolic subbands. The peaks positions arises from the band edge state energies and the density of states heights are proportional to inverse square root of the sub band curvature and band degeneracy. For determining the chemical potential, $\mu$, we use the relation between concentration of electrons ($n_{e}$) and chemical potential. This relation is given by

17 $$\begin{aligned} n_{e}=\frac{1}{4N}\sum _{\mathbf{k},\eta ,\sigma }\frac{1}{e^{E^{\sigma }_{\eta }(\mathbf{k})/k_{B}T}+1}. \end{aligned}$$

Based on the values of electronic concentration $n_{e}$, the chemical potential, $\mu$, can be obtained by means Eq. (17).

## Theoretical calculation of electrical conductivity, thermal conductivity and thermoelectric properties

Using linear response theory, the thermal conductivity under the assumption of a low temperature gradient (as a perturbing field) is obtained. The charge and thermal current are related to the gradients $\nabla V$, which is equal to external electric current $\mathbf{E}$ and $\nabla T$ of the electric potential and the temperature^35^ ,respectively, by

18 $$\begin{aligned} \left( \begin{array}{cccc} \mathbf{J}_{1}\\ \mathbf{J}_{2} \\ \end{array} \right) =\left( \begin{array}{cccc} L_{11} & L_{12} \\ L_{21} & L_{22} \\ \end{array} \right) \left( \begin{array}{cccc} -\mathbf{E}=\nabla V\\ -\mathbf{\nabla } T\\ \end{array} \right) . \end{aligned}$$

$\mathbf{J}_{1(2)}=\mathbf{J}_{e}(\mathbf{J}_{Q})$ implies electrical (heat) current. Also $L_{ab}(a,b=1,2)$ are transport coefficients which are determined by calculating correlation function between the electrical and thermal current operators. The thermal conductivity is obtained as the response of the heat current ($\mathbf{{J}}_{Q}$) to a temperature gradient. Imposing the continuity equation for the energy density, $\frac{\partial }{\partial t}H+\nabla \cdot \mathbf{J} _{Q}=0$, the explicit form of the heat current can be calculated. Using the continuity equations for charge and heat, the thermal and electrical current operators, i.e. $\mathbf{{J}}_{Q}$ and $\mathbf{{J}}_{e}$ for itinerant electrons in the context of Kane–Mele model are reduced to^35^

19 $$\begin{aligned} \mathbf{{J}}_{Q}=\sum _{\mathbf{k},\sigma ,\eta =1,2,3} \mathbf{v}_{\eta }(\mathbf{k})E^{\sigma }_{\eta }(\mathbf{k})c^{\dag \sigma } _{\eta ,\mathbf{k}}c^{\sigma }_{\eta ,\mathbf{k}}\;\;,\;\;\mathbf{{J}}_{e}=\sum _{\mathbf{k},\sigma ,\eta =1,2,3} \mathbf{v}_{\eta }(\mathbf{k})c^{\dag \sigma } _{\eta ,\mathbf{k}}c^{\sigma }_{\eta ,\mathbf{k}}, \end{aligned}$$

so that $\mathbf{v}_{\eta }(\mathbf{k})=\mathbf {\nabla }_\mathbf{k}E^{\sigma }_{\eta }(\mathbf{k})$ denotes the group velocity of bosonic particles in electronic band structure index $\eta$. The linear response theory is implemented to obtain the thermal conductivity under the assumption of a low temperature gradient (as a perturbing field). The Kubo formula gives the transport coefficients $L^{xx}_{22}(\omega )$ in terms of a correlation function of energy current operators^35^

20 $$\begin{aligned} L^{xx}_{22}(\omega )= &  -\mathrm Im\frac{ik_{B}T}{\omega }\int _{-\infty }^{+\infty }dt e^{i\omega t} \theta (t)\langle [J^{{\rm x}}_{Q}(t),J^{{\rm x}}_{Q}(0)]\rangle \nonumber \\ = &  -\mathrm Im \frac{k_{B}T}{\omega }\lim _{i\omega _{n}\longrightarrow \omega +i0^{+}} \int ^{1/(k_{B}T)}_{0}d\tau e^{i\omega _{n}\tau }\langle T_{\tau }(J^{{\rm x}}_{Q}(\tau ) J^{{\rm x}}_{Q}(0))\rangle , \end{aligned}$$

where it is assumed that energy current flows along zigzag direction( i.e. *x*). The energy current density is related to the temperature gradient via $\mathbf{J}_{Q}=-K\nabla T$ where *K* is the thermal conductivity^35,36^. We calculate the correlation function in Eq. (20) within an approximation by implementing Wick’s theorem. The correlation functions between current operators can be written as

21 $$\begin{aligned} \langle T_{\tau }(J^{x}_{Q}(\tau )J^{x}_{Q}(0))\rangle= &  \sum _{\mathbf{k},\mathbf{k}',\eta ,\eta '} v^{x}_{\eta }(\mathbf{k})v^{x}_{\eta '}(\mathbf{k'}) E^{\sigma }_{\eta }(\mathbf{k}) E^{\sigma }_{\eta '}(\mathbf{k'})\langle T(c^{\sigma \dag }_{\eta ,\mathbf{k}}(\tau )c^{\sigma }_{\eta ,\mathbf{k}}(\tau ) c^{\sigma '\dag }_{\eta ',\mathbf{k}'}(0)c^{\sigma '}_{\eta ',\mathbf{k}'}(0))\rangle . \end{aligned}$$

Applying the Wick’s theorem leads to the following expression for energy current correlation function as

22 $$\begin{aligned} \langle T_{\tau }(J^{x}_{Q}(\tau )J^{x}_{Q}(0))\rangle =\sum _{\mathbf{k},\eta ,\sigma } (v^{x}_{\eta }(\mathbf{k}))^{2} E_{\eta }^{2}(\mathbf{k})G^{\sigma }_{\eta }(\mathbf{k},\tau )G^{\sigma }_{\eta }(\mathbf{k},-\tau ). \end{aligned}$$

By substituting Eq. (22) into Eq. (20) and using Fourier transformation of bosonic Green’s function, i.e. $G^{\sigma }_{\eta }(\mathbf{k},\tau )=k_{B}T\sum _{m}e^{-i\omega _{m}\tau }G^{\sigma }_{\eta }(\mathbf{k},i\omega _{m})$, transport coefficient $L^{xx}_{22}(\omega )$ can be expressed in terms of fermionic Green’s function as

23 $$\begin{aligned} L^{xx}_{22}(\omega )= &  \frac{k_{B}T}{\omega }\lim _{i\omega _{n}\longrightarrow \omega +i0^{+}} \int ^{1/k_{B}T}_{0}d\tau e^{i\omega _{n}\tau }\langle T_{\tau }(J^{x}_{Q}(\tau )J^{x}_{Q}(0)) \rangle \nonumber \\= &  \frac{(k_{B}T)^{2}}{\omega }\lim _{i\omega _{n}\longrightarrow \omega +i0^{+}} \sum _{\mathbf{k},\eta ,\sigma }\sum _{m}\Big ((v^{x}_{\eta }(\mathbf{k}) E^{\sigma }_{\eta }(\mathbf{k}))^{2}G^{\sigma }_{\eta }(\mathbf{k},i\omega _{m})G^{\sigma }_{\eta }(\mathbf{k},i\omega _{n}+i\omega _{m})\Big ). \end{aligned}$$

According to the Lehmann representation^35^, the imaginary part of retarded Green’s function and Matsubara form of Green’s function are related to each other as

24 $$\begin{aligned} G^{\sigma }_{\eta }(\mathbf{k},i\omega _{m})=\int ^{+\infty }_{-\infty }\frac{d\epsilon }{2\pi } \frac{-2 Im\Big (G^{\sigma }_{\eta } (\mathbf{k},\epsilon +i0^{+})\Big )}{i\omega _{m}-\epsilon }, \end{aligned}$$

Using Lehmann representation, the expression for transport coefficient $L^{xx}_{22}(\omega )$ in Eq. (23) is given by

25 $$\begin{aligned} L^{xx}_{22}(\omega )= &  \frac{(k_{B}T)^{2}}{\omega } \lim _{i\omega _{n}\longrightarrow \omega +i0^{+}} \sum _{\mathbf{k},\eta ,\sigma }\sum _{m}\int ^{+\infty }_{-\infty }\frac{d\epsilon }{2\pi } \int ^{+\infty }_{-\infty }\frac{d\epsilon '}{2\pi } (v^{x}_{\eta }(\mathbf{k})E^{\sigma }_{\eta }(\mathbf{k}))^{2} \nonumber \\ \times &  2 Im\Big (G^{\sigma }_{\eta } (\mathbf{k},\epsilon +i0^{+})\Big )2 Im\Big (G^{\sigma }_{\eta } (\mathbf{k},\epsilon '+i0^{+})\Big ) \times \frac{1}{i\omega _{m}-\epsilon } \frac{1}{i\omega _{n}+i\omega _{m}-\epsilon '}. \end{aligned}$$

After summation over Matsubara’s bosonic frequency $\omega _{m}$ the result form for $L^{xx}_{22}(\omega )$ is given by

26 $$\begin{aligned} L^{xx}_{22}(\omega )= &  \frac{k_{B}T}{\omega } \lim _{i\omega _{n}\longrightarrow \omega +i0^{+}} \sum _{\mathbf{k},\eta ,\sigma }\int ^{+\infty }_{-\infty }\frac{d\epsilon }{2\pi } \int ^{+\infty }_{-\infty }\frac{d\epsilon '}{2\pi } (v^{x}_{\eta }(\mathbf{k})E^{\sigma }_{\eta }(\mathbf{k}))^{2} \nonumber \\ \times &  2 Im\Big (G^{\sigma }_{\eta } (\mathbf{k},\epsilon +i0^{+})\Big )2 Im\Big (G^{\sigma }_{\eta } (\mathbf{k},\epsilon '+i0^{+})\Big ) \frac{n_{F}(\epsilon )-n_{F}(\epsilon ')}{i\omega _{n}+\epsilon -\epsilon '}. \end{aligned}$$

Using Eqs. (20 and 26), we can derive the following relation for static transport coefficient $L^{xx}_{22}$ of localized electrons on Lieb lattice as

27 $$\begin{aligned} L_{22}^{xx}=\mathrm{lim}_{\omega \longrightarrow 0}L^{xx}_{22}(\omega ) =-k_{B}T\sum _{\mathbf{k},\eta ,\sigma }\int ^{+\infty }_{-\infty }\frac{d\epsilon }{2\pi } (v^{x}_{\eta }(\mathbf{k})E^{\sigma }_{\eta }(\mathbf{k}))^{2} \Big (Im(G^{\sigma }_{\eta } (\mathbf{k},\epsilon +i0^{+}))\Big )^{2}\frac{dn_{F}(\epsilon )}{d\epsilon }. \end{aligned}$$

where $n_{F}(x)=\frac{1}{e^{x/k_{B}T}+1}$ is the Fermi-Dirac distribution function and *T* denotes the equilibrium temperature. In Appendix B, we have presented the explicit relations for the other transport coefficients $L^{xx}_{11},L^{xx}_{12}$.

Substituting electronic Green’s function into Eqs. (27 and 33) and performing the numerical integration over wave vector through first Brillouin zone, the results of transport coefficients have been obtained. The electrical conductivity $\sigma$ is usually defined when is no temperature gradient $\nabla T=0$. Thus we have

28 $$\begin{aligned} \sigma =\frac{L_{11}^{xx}}{k_{B}T} \end{aligned}$$

In the presence of a temperature gradient ($\nabla T$) and in open circuit situation, i.e.$\mathbf{J}^{e}=0$, heat current is related to temperature gradient via $\mathbf{J}^{Q}=K\nabla T$ where $\kappa$ is the thermal conductivity and is obtained using transport coefficients as^35^

29 $$\begin{aligned} \kappa= &  \frac{1}{T^2}(L_{22}-\frac{L_{12}^{2}}{L_{11}}). \end{aligned}$$

Applying a temperature gradient to Lieb lattice and under no particle current situation $\mathbf{J}_{e}=0$, a voltage difference $\Delta V$ can be measured which is proportional to $\Delta T$. The ratio of the measured voltage to the temperature gradient applied across the sample is known as the Seebeck coefficient (or the thermopower) and is given by $S=\nabla V/\nabla T$, where $\nabla V$ is the potential difference between two points of the sample^36^. In linear response approximation the Seebeck coefficient is related to transport coefficients as

30 $$\begin{aligned} S=-\frac{1}{T}\frac{L_{12}}{L_{11}} \end{aligned}$$

The study of behaviors of thermal conductivity $\kappa$, Seebeck coefficient S and electrical conductivity $\sigma$ in two dimensional Lieb lattice in the presence of magnetic field and spin-orbit coupling is the main aim in this work.

## Numerical results and discussion

Here we present our numerical results for the transport and thermoelectric properties of single layer Lieb lattice in the presence of magnetic field and spin-orbit coupling effects. We focus on the electrical conductivity, Seebeck coefficient and thermal conductivity of doped Lieb lattice in the presence of both magnetic field and spin-orbit coupling. We have studied the transport properties of the structure along *x* direction according to Fig. 1. Using band structure of electron in Eq. (31), we can obtain the electronic Green’s function in Eq. (15). Afterwards *x* components of transport coefficients $L_{11}, L_{12} ,L_{22}$ are found by substitution of Green’s function into Eqs. (27 and 33), respectively. The numerical results of electrical conductivity $\sigma$, thermal conductivity $\kappa$ and Seebeck coefficient *S* can be found using Eqs. (33, 29 and 30). The optimized atomic structure of the Lieb lattice with primitive unit cell vector length $a=1$ is shown in Fig. 1. The primitive unit cell include three different atoms with various on-site energies.

We begin the investigations of thermal properties of Lieb lattice layer with a discussion of the thermal conductivity in the presence of magnetic field and spin-orbit coupling effects. In left panel of Fig. 2 results for the thermal conductivity ($\kappa (T)$) of doped Lieb lattice are presented versus normalized temperature ($k_{B}T/t$, where $k_{B}$ is the Boltzmann constant) for different gap parameter values in the absence of magnetic field. The normalized chemical potential and spin-orbit coupling strength are assumed to be $\mu /t=1.0$ and $\lambda /t=0.2$ respectively. Here we have pointed some arguments regarding the normalized values for $\mu /t=1.0$ and $\lambda /t=0.2$. We have studied the effects of spin-orbit coupling values on the transport properties of Lieb lattice. Also chemical potential $\mu$ which is related to electronic concentration. In fact there is no special physical reason for these values for chemical potential and spin-orbit coupling effects. $\mu$ and $\lambda$ are parameters and we can change their values for investigation of transport properties of the structure. Since we have assumed $t=1$, we have introduced the normalized values for $\mu$ and $\lambda$ for instance 1.0 and 0.2, respectively. However we have considered the other values for these parameters in the next results. Several features are remarkable. Each curve shows an increasing behavior at low temperatures which manifests the presence of a finite-energy gap in the energy spectrum. There is also a finite temperature maximum in the thermal conductivity so that its temperature position is around normalized value $k_{B}T/t=0.4$ for gap parameter values $\Delta /t=0.3,0.4,0.5,0.6,0.7$. In addition, at fixed value of temperature on the whole range of temperature, thermal conductivity of Lieb lattice increases with normalized gap parameter. Below the characteristic temperature of the maximum, the enhancement of temperature leads to increase the rate of transition of electrons to the excited state. Therefore we see an increasing behavior for the thermal conductivity at low temperatures, see left panel of Fig. 2. With increasing temperature above peak position, the electrons suffer from scattering effects on each other which reduces the thermal conductivity. Hence the temperature dependence of each curve is due to competition between the two phenomena, the **enhancement** of the transition rate of electrons from ground state to excited one within the physical limits and the scattering of the electrons at higher temperatures.

**Figure 3:**
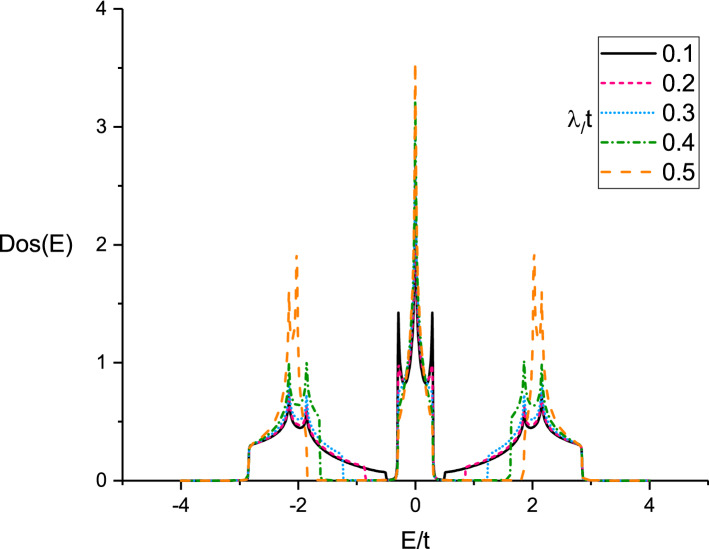
Density of states (DOS(E)) of electrons on Lieb lattice as a function of energy for different values of normalized spin-orbit coupling strength $\lambda /t$ in the absence of magnetic field at fixed gap parameter $\Delta /t=0.3$.

The temperature behavior of thermal conductivity of doped Lieb lattice for different spin-orbit coupling strength has been plotted in right panel of Fig. 2. We have assumed chemical potential as $\mu /t=1.0$ in the absence of magnetic field at fixed gap parameter $\Delta /t=0.3$. Thermal conductivity curve for each spin-orbit coupling value includes a peak so that the peak appears at normalized value $k_{B}T/t=0.7$ which is independent of $\lambda$. Thermal conductivity for each value of spin-orbit coupling increases with temperature up to temperature position of peak due to **enhancement** of transition rate of electrons between quantum levels. **With** increasing temperature above peak position, thermal conductivity decreases with temperature which understood based on enhancement of scattering rate between electrons and consequently the conductivity reduces with temperature above temperature position of peak. In addition, at fixed temperature, we find thermal conductivity reduces with **enhancement** of $\lambda$ as shown in right panel of Fig. 2. The numerical results of density of states show the band gap width in density of states rises with $\lambda$ which leads to reduce thermal conductivity. The energy dependence of density of states and the increase of band gap in density of states due to spin-orbit coupling strength has been shown in Fig. 3. Based on Fig. 3, the band gap around normalized chemical potential $E/t=\mu /t=1$ enhances with $\lambda /t$.

**Figure 4:**
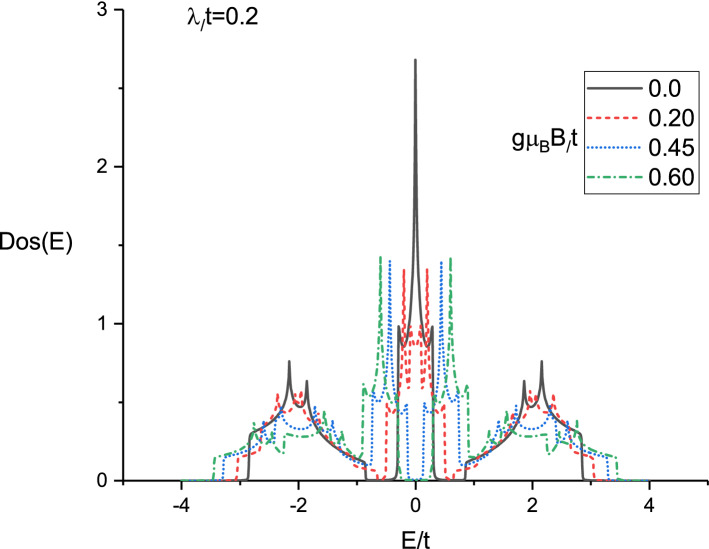
Density of states (DOS(E)) of electrons on Lieb lattice as a function of energy for different values of normalized magnetic field strength ,namely $g\mu _{B}B/t=0.0,0.20,0.45,0.60$ for $\lambda /t=0.2$ at fixed gap parameter $\Delta /t=0.3$.

**Figure 5:**
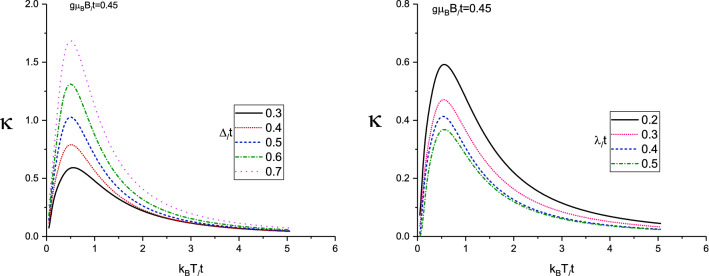
Left Panel: Thermal conductivity of Lieb lattice as a function of normalized temperature $k_{B}T/t$ for different values of normalized gap parameter $\Delta /t$ in the presence of magnetic field $g\mu _{B}B/t=0.45$. Normalized chemical potential and normalized spin-orbit coupling have been fixed at $\mu /t=1.0$ and $\lambda /t=0.2$, respectively. Right Panel: Thermal conductivity of Lieb lattice as a function of normalized temperature $k_{B}T/t$ for different values of normalized spin-orbit coupling $\lambda /t$ in the presence of magnetic field $g\mu _{B}B/t=0.45$. Normalized chemical potential and normalized gap parameter have been fixed at $\mu /t=1.0$ and $\Delta /t=0.3$, respectively.

In left panel of Fig. 5 thermal conductivity results of doped Lieb lattice are presented versus normalized temperature $k_{B}T/t$ for different gap parameter values at fixed magnetic field $g\mu _{B}B/t=0.45$. Such magnetic field value introduces the critical value where the transition from **metallic** phase to semiconductor one takes place. The normalized chemical potential and spin-orbit coupling strength are assumed to be $\mu /t=1.0$ and $\lambda /t=0.2$ respectively. **Such critical value** $g\mu _{B}B/t=0.45$ **can be found based on the behavior of density of states of Lieb lattice for different magneic fields in Fig.** 4. **Based on this figure we have found that a band gap appears in density of states at critical magnetic field** $g\mu _{B}B/t=0.45$.

As it is clearly observed in left panel of Fig. 5, each curve shows an increasing behavior at low temperatures which manifests the presence of a finite-energy gap in the energy spectrum. There is also a finite temperature maximum in the thermal conductivity so that its temperature position is around normalized value $k_{B}T/t=0.4$ for gap parameter values $\Delta /t=0.3,0.4,0.5,0.6,0.7$. In addition, at fixed value of temperature on the whole range of temperature, thermal conductivity of Lieb lattice **enhances** with normalized gap parameter. The enhancement of temperature below peak position leads to increase the rate of transition of electrons to the excited state and consequently enhancement of the thermal conductivity in temperature region below peak position. **With more increasing the temperature**, the electrons suffer from scattering effects on each other which reduces the thermal conductivity.

The temperature behavior of thermal conductivity of doped Lieb lattice for different spin-orbit coupling strength for $\mu /t=1.0$ at fixed gap parameter $\Delta /t=0.3$ has been plotted in right panel of Fig. 5. The applied magnetic field strength has been considered to be critical value $g\mu _{B}B/t=0.45$. Such critical magnetic field value describes the phase transition form metal to semiconductor in Lieb lattice. Thermal conductivity curve for each spin-orbit coupling value has a peak so that the peak appears at normalized value $k_{B}T/t=0.7$ which is independent of $\lambda$. In addition, at fixed temperature, we find the decreasing behavior for thermal conductivity in terms of $\lambda$ as shown in right panel of Fig. 5. Such fact is understood from this point that density of states of Lieb lattice show the band gap width in density of states increases with $\lambda$ which leads to reduce thermal conductivity. The appearance of peak in temperature dependence of each curve is due to competition between two phenomena, the enhancement of the transition rate of electrons from ground state to excited one within the physical limits and the scattering of the electrons at higher temperatures.

**Figure 6:**
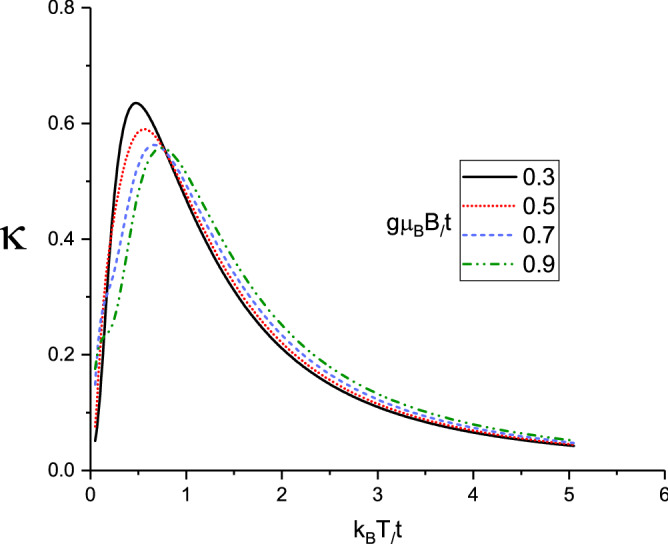
Thermal conductivity of Lieb lattice as a function of normalized temperature $k_{B}T/t$ for different values of normalized magnetic field $g\mu _{B}B/t$ at fixed gap parameter $\Delta /t=0.3$. Normalized chemical potential and normalized spin-orbit coupling strength have been fixed at $\mu /t=1.0$ and $\lambda /t=0.2$, respectively.

We have also studied the effect of the magnetic field values, i.e. $g\mu _{B}B/t$, on the temperature dependence of the thermal conductivity of the doped Lieb lattice. In Fig. 6 we plot $\kappa$ versus normalized temperature for several values of the longitudinal magnetic field, namely $g\mu _{B}B/t=0.3,0.5,0.7,0.9$. The spin-orbit coupling and gap parameter have been fixed at $\lambda /t=0.2$ at $\Delta /t=0.3$, respectively. The normalized chemical potential value has been considered at $\mu /t=1.0$. Chemical potential values above zero mean the average of number of electrons per atomic site is higher than unit. In other words the electronic concentration is above one. This plot shows that thermal conductivities enhances with reduction of the magnetic field at fixed temperature value in temperature region $k_{B}T/t<1.0$. However thermal conductivity rises with magnetic field at fixed temperature in the regions $k_{B}T/t>1.0$. The height of peak in thermal conductivity reduces with magnetic field although the temperature position of peak moves to higher amounts as shown in Fig. 6.

**Figure 7:**
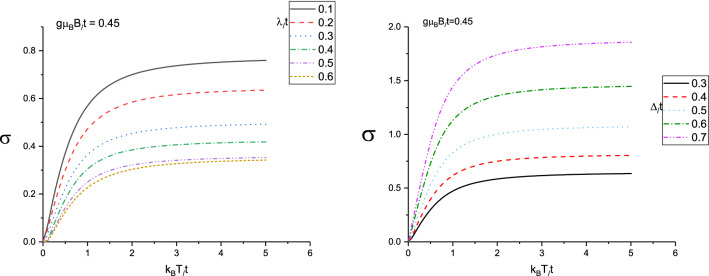
Left Panel: Electrical conductivity of Lieb lattice as a function of normalized temperature $k_{B}T/t$ for different values of normalized spin-orbit coupling $\lambda /t$ in the presence of magnetic field $g\mu _{B}B/t=0.45$. Normalized chemical potential and normalized gap parameter have been fixed at $\mu /t=1.0$ and $\Delta /t=0.3$, respectively. Right Panel:Electrical conductivity of Lieb lattice as a function of normalized temperature $k_{B}T/t$ for different values of normalized gap parameter $\Delta /t$ in the presence of magnetic field $g\mu _{B}B/t=0.45$. Normalized chemical potential and normalized spin-orbit coupling have been fixed at $\mu /t=1.0$ and $\lambda /t=0.2$, respectively.

We have studied the static electrical conductivity of doped Lieb lattice layer due to magnetic field and spin-orbit coupling strength. The resulting of the electrical conductivity $\sigma$ of doped Lieb lattice layer as a function of normalized temperature $k_{B}T/t$ for different values of spin-orbit coupling constant at $g\mu _{B}B/t=0.45$ has been plotted in left panel of Fig. 7. The applied magnetic field strength has been considered to be critical value $g\mu _{B}B/t=0.45$. The normalized gap parameter and chemical potential are assumed to be $\Delta /t=0.3$ and $\mu /t=1.0$ respectively. This figure indicates the increasing behavior for temperature dependence of electrical conductivity is clearly observed in temperature region $k_{B}T/t<1.0$ for all amounts of $\lambda /t$. **With increase of temperature above normalized value 1.0, the electrical conductivity gets a constant value**. In addition, at fixed value of normalized temperature, lower spin-orbit coupling causes less band gap in density of states and consequently higher values in electrical conductivity as shown in left panel of Fig. 7.

The resulting of the electrical conductivity $\sigma$ of doped Lieb lattice layer as a function of normalized temperature $k_{B}T/t$ for different normalized values of gap parameter at $g\mu _{B}B/t=0.45$ has been plotted in right panel of Fig. 7. The chemical potential normalized value has been considered to be $\mu /t=1.0$. This figure indicates the increasing behavior for temperature dependence of electrical conductivity is clearly observed in temperature region $k_{B}T/t<1.0$ for all amounts of gap parameters. The enhancement of temperature above normalized value 1.0, the electrical conductivity gets a constant value. The increase of gap parameter in the presence of spin-orbit coupling leads to reduce band gap in density of states. Thus higher gap parameter causes less band gap in density of states and consequently higher values in electrical conductivity at fixed value of normalized temperature.

**Figure 8:**
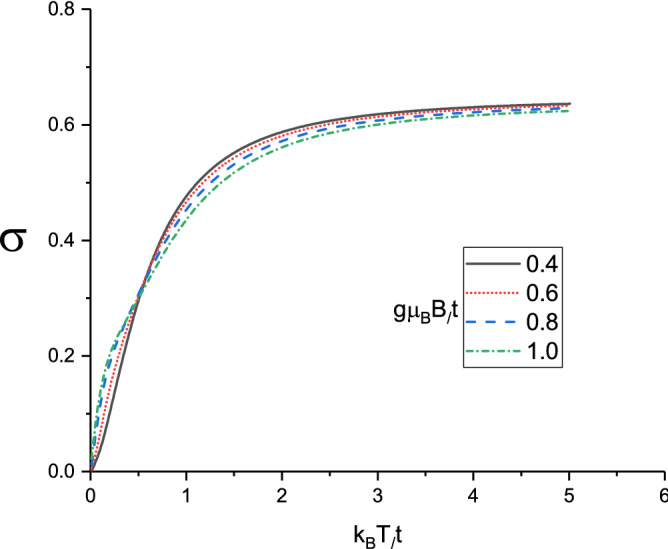
Electrical conductivity of Lieb lattice as a function of normalized temperature $k_{B}T/t$ for different values of normalized magnetic field $g\mu _{B}B/t$ at fixed gap parameter $\Delta /t=0.3$. Normalized chemical potential and normalized spin-orbit coupling strength have been fixed at $\mu /t=1.0$ and $\lambda /t=0.2$, respectively.

In Fig. 8 we present the electrical conductivity of doped Lieb lattice versus normalized temperature, $k_{B}T/t$ for different values of magnetic field, namely $g\mu _{B}B/t=0.4,0.6,0.8,1.0$.The chemical potential and spin-orbit coupling strength have been assumed to be $\mu /t=1.0$ and $\lambda /t=0.2$, respectively. Also the normalized gap parameter is considered to be $\Delta /t=0.3$. According to Fig. 8, the electrical conductivity shows an increasing behavior with temperature in the region $k_{B}T/t<2.0$. **With increasing temperature **above normalized value 0.9, the conductivity is less temperature dependent for each value of magnetic field. Moreover at fixed temperature below normalized value 0.6, electrical conductivity rises with magnetic field. However the conductivity reduces with magnetic field at fixed temperature above normalized value 0.6 as shown in Fig. 8.

**Figure 9:**
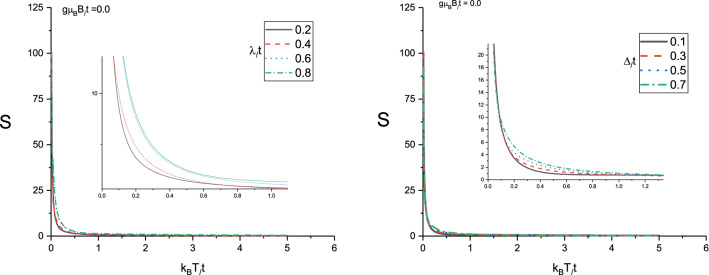
Left panel: Seebeck coefficient of Lieb lattice as a function of normalized temperature $k_{B}T/t$ for different values of normalized spin-orbit coupling $\lambda /t$ in the absence of magnetic field. Normalized chemical potential and normalized gap parameter have been fixed at $\mu /t=1.0$ and $\Delta /t=0.3$, respectively. Right Panel: Seebeck coefficient of Lieb lattice as a function of normalized temperature $k_{B}T/t$ for different values of normalized gap parameter $\Delta /t$ in the absence of magnetic field. Normalized chemical potential and normalized spin-orbit coupling have been fixed at $\mu /t=1.0$ and $\lambda /t=0.2$, respectively.

Considering magneto thermal effects using Eq. (18), the Seebeck coefficient *S* under the condition of zero electrical current $\mathbf{J}^{e}=0$ and for ballistic transport is given by Eq. (30). In left panel of Fig. 9 we depict the Seebeck coefficient *S* of doped monolayer Lieb lattice as a function of normalized temperature $k_{B}T/t$ for several values of normalized spin-orbit coupling $\lambda /t$ at zero magnetic field with $\mu /t=1.0$. The normalized gap parameter is assumed to be 0.3. We note that the variation of spin orbit coupling strength has no considerable effect on temperature dependence of Seebeck coefficient of Lieb lattice layer. A monotonic decreasing behavior for Seebeck coefficient is clearly observed for each value of spin-orbit coupling strength according to left panel of Fig. 9. In Ref.(^37^), it was suggested that the sign of *S* is a criterion to clarify the types of carriers; a positive (negative) *S* implies that the charge and heat are dominantly carried by electrons (holes). Also Seebeck coefficient curves in left panel of Fig. 9 gets the positive sign for Seebeck coefficient on the whole range of temperature for all values of spin-orbit coupling and consequently the majority of charge carriers are holes. The inset in left panel of Fig. 9 focuses on the behavior of *S* for $k_{B}T/t<1.0$. Left panel of Fig. 9 indicates the Seebeck coefficient tends to zero for all $\lambda /t$ at temperatures above normalized value 2.0.

The behavior of Seebeck coefficient *S* of doped Lieb lattice layer as a function of normalized temperature $k_{B}T/t$ for different normalized gap parameter $\Delta /t$ at fixed spin-orbit coupling $\lambda /t=0.2$ in the absence of applied magnetic field has been plotted in right panel of Fig. 9. The chemical potential is considered to be $\mu /t=1.0$. A monotonic decreasing for temperature dependence of Seebeck coefficient is observed for each value of gap parameter. However Seebeck coefficient rises with gap parameter at fixed temperature in the region $0.2<k_{B}T/t<1.0$ as shown in the inset in right panel of Fig. 9. Moreover the sign of Seebeck coefficient for all values of temperature and gap parameter amounts is positive.

**Figure 10:**
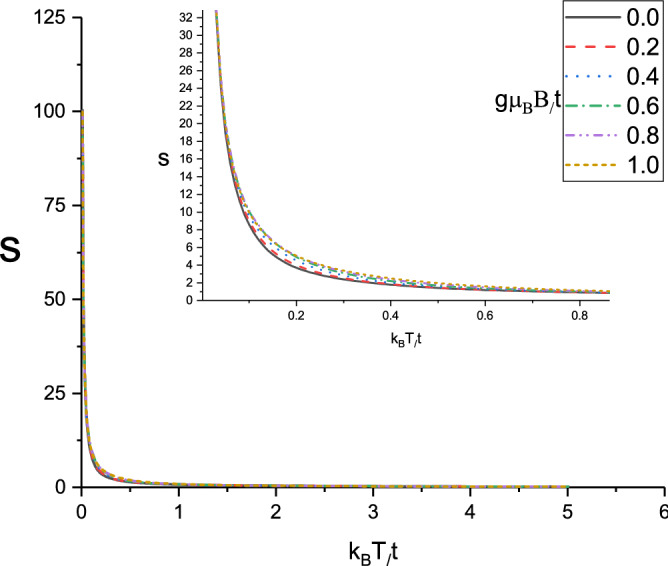
Seebeck coefficient of Lieb lattice as a function of normalized temperature $k_{B}T/t$ for different values of normalized magnetic field $g\mu _{B}B/t$ at fixed gap parameter $\Delta /t=0.3$. Normalized chemical potential and normalized spin-orbit coupling strength have been fixed at $\mu /t=1.0$ and $\lambda /t=0.2$, respectively.

The dependence of Seebeck coefficient of doped Lieb lattice layer on the temperature for different magnetic fields, namely $g\mu _{B}B/t=0.0,0.2,0.4,0.6,0.8,1.0$ at normalized spin-orbit coupling $\lambda /t=0.2$ has been plotted in Fig. 10. The chemical potential and gap parameter have been fixed at normalized values $\mu /t=1.0$ and $\Delta /t=0.3$ respectively. The sign of Seebeck coefficient is positive for all values of magnetic fields on the whole range of temperature. It is clearly observed that there is a monotonic decreasing temperature dependence for all magnetic fields. In addition, at fixed normalized temperature in the region $0.1<k_{B}T/t<0.4$, Seebeck coefficient **grows** with magnetic field. However the curves of Seebeck coefficient for different values of magnetic fields fall on each other at temperatures above normalized value 0.6 according to Fig. 10. Thermoelectric properties of semimetallic low dimensional structures and Lieb lattice have been reported in the other studies^38,39^. Also the effects of strains on electronic structure and thermoelectric properties of half-Heusler semiconductor^40^.

## Conclusions

In summary, we have studied the thermal transport properties of Lieb lattice in the presence of magnetic field and intrinsic spin-orbit coupling strength. Also the difference between on-site energies of atoms on three different sublattices, as a gap parameter, has been considered. The Lieb lattice is an interesting lattice model related to magnetism. Also the Lieb lattice exhibits the interesting transport properties, superconducting transitions, magnetic phase transition and quantum spin Hall effect. Such properties make the motivation for studying the electronic properties of this structure. This lattice has been applied to describe the CuO$_{2}$ planes of cuprate superconductors. When the intrinsic spin-orbit coupling is introduced to the Lieb lattice, a topological insulator can be developed so that the structure shows an insulating behavior in the bulk and metallic property in the surfaces and edges. Such coupling gives rise to the quantum spin Hall effect. Our main purpose for this present study is the investigation of transport properties and thermoelectric features for Lieb lattice in the presence of spin-orbit coupling. In the absence of magnetic field, Lieb lattice shows a metallic behavior due to the competition between gap parameter and spin-orbit coupling strength parameters. **With enhancement of magnetic field above critical one**, a phase transition from metallic phase to insulating behavior at critical value $g\mu _{B}B/t=0.45$ for all values of spin-orbit coupling strength takes place. The temperature dependence of thermal conductivity for different gap parameters in the absence of magnetic field shows that there is a peak in thermal conductivity curves so that the height of peak increases with gap parameter. Thermal conductivity results of Lieb lattice in the presence of critical magnetic field $g\mu _{B}B/t=0.45$ for different gap parameters indicate the similar behaviors with zero magnetic field case. Also we have studied the effects of spin-orbit coupling strength on temperature dependence of thermal conductivity in the presence of critical magnetic field $g\mu _{B}B/t=0.45$. Our results show the height of peak in thermal conductivity decreases with spin-orbit coupling strength. The effects of spin-orbit coupling on density of states leads to increase band gap so that thermal conductivity values reduces. The appearance of peak in temperature dependence of thermal conductivity as a competition between two physical phenomena is clearly observed in our results. Also the numerical results for thermopower which its signature determines the type of majority change carriers have been obtained. Moreover the decreasing behavior of Seebeck coefficient in terms of temperature is the other novel results in our study. Our numerical results for Seebeck coefficient of Lieb lattice show that the variation of spin-orbit coupling strength and gap parameter have no effect on the sign of Seebeck coefficient. In other words the Seebeck coefficient gets positive sign under the variations of spin-orbit coupling, gap parameter and temperature. This fact implies the holes play the role of the majority carriers for transport in Lieb lattice.

## Appendix A: The eigenvalues of total Hamiltonian in Eq. (11)

In this Appendix, we have presented the explicit relations for eigenvalues of total Hamiltonian in Eq. (11), After diagonalizing of the Hamiltonians in Eq. (11), the band structures of Lieb lattice are given by following eigenvalues

31 $$\begin{aligned} E_{1}^{\sigma }(\mathbf{k})= &  \frac{2}{\sqrt{3}}\sqrt{\Delta ^{2}+\chi ^{2}_{x}+\chi ^{2}_{y}+\chi ^{2}}\cos \Big (\frac{1}{3}\sin ^{-1} (\frac{3\sqrt{3}\Delta (\chi ^{2}_{y}-\chi ^{2}_{x})}{2(\Delta ^{2}+\chi ^{2}_{x}+\chi ^{2}_{y}+\chi ^{2})^{3/2}})+\pi /6\Big )-\mu -\sigma g\mu _{B}B, \nonumber \\ E_{2}^{\sigma }(\mathbf{k})= &  \frac{2}{\sqrt{3}}\sqrt{\Delta ^{2}+\chi ^{2}_{x}+\chi ^{2}_{y}+\chi ^{2}}\sin \Big (\frac{1}{3}\sin ^{-1} (\frac{3\sqrt{3}\Delta (\chi ^{2}_{y}-\chi ^{2}_{x})}{2(\Delta ^{2}+\chi ^{2}_{x}+\chi ^{2}_{y}+\chi ^{2})^{3/2}})\Big )-\mu -\sigma g\mu _{B}B,\nonumber \\ E_{3}^{\sigma }(\mathbf{k})= &  -\frac{2}{\sqrt{3}}\sqrt{\Delta ^{2}+\chi ^{2}_{x}+\chi ^{2}_{y}+\chi ^{2}}\sin \Big (\frac{1}{3}\sin ^{-1} (\frac{3\sqrt{3}\Delta (\chi ^{2}_{y}-\chi ^{2}_{x})}{2(\Delta ^{2}+\chi ^{2}_{x}+\chi ^{2}_{y}+\chi ^{2})^{3/2}})+\pi /3\Big )-\mu -\sigma g\mu _{B}B.\nonumber \\ \end{aligned}$$

## Appendix B: The explicit relations for transport coefficients $L_{12}, L_{11}$

Also the other transport coefficients are defined by

32 $$\begin{aligned} L^{xx}_{11}(\omega )= &  -\mathrm Im\frac{ik_{B}T}{\omega }\int _{-\infty }^{+\infty }dt e^{i\omega t} \theta (t)\langle [J^{x}_{e}(t),J^{x}_{e}(0)]\rangle \nonumber \\ = &  -\mathrm Im \frac{k_{B}T}{\omega }\lim _{i\omega _{n}\longrightarrow \omega +i0^{+}} \int ^{1/(k_{B}T)}_{0}d\tau e^{i\omega _{n}\tau }\langle T_{\tau }(J^{x}_{e}(\tau ) J^{x}_{e}(0))\rangle ,\nonumber \\ L^{xx}_{12}(\omega )= &  -\mathrm Im\frac{ik_{B}T}{\omega }\int _{-\infty }^{+\infty }dt e^{i\omega t} \theta (t)\langle [J^{x}_{e}(t),J^{x}_{Q}(0)]\rangle \nonumber \\ = &  -\mathrm Im \frac{k_{B}T}{\omega }\lim _{i\omega _{n}\longrightarrow \omega +i0^{+}} \int ^{1/(k_{B}T)}_{0}d\tau e^{i\omega _{n}\tau }\langle T_{\tau }(J^{x}_{e}(\tau ) J^{x}_{Q}(0))\rangle \end{aligned}$$

After some calculations similar to static transport coefficient $L^{xx}_{22}$, we can express transport coefficients $L^{xx}_{11}$ and $L^{xx}_{12}$ in terms of electronic Green’s function as following relations

33 $$\begin{aligned} L_{11}^{xx}=\, &  \mathrm{lim}_{\omega \longrightarrow 0}L^{xx}_{11}(\omega ) =-k_{B}T\sum _{\mathbf{k},\eta ,\sigma }\int ^{+\infty }_{-\infty }\frac{d\epsilon }{2\pi } \Big (v^{x}_{\eta }(\mathbf{k})\Big )^{2} \Big (Im(G^{\sigma }_{\eta } (\mathbf{k},\epsilon +i0^{+}))\Big )^{2}\frac{dn_{F}(\epsilon )}{d\epsilon },\nonumber \\ L_{12}^{xx}=\, &  \mathrm{lim}_{\omega \longrightarrow 0}L^{xx}_{12}(\omega ) =-k_{B}T\sum _{\mathbf{k},\eta ,\sigma }\int ^{+\infty }_{-\infty }\frac{d\epsilon }{2\pi } \Big (v^{x}_{\eta }(\mathbf{k})\Big )^{2}E^{\sigma }_{\eta }(\mathbf{k}) \Big (Im(G^{\sigma }_{\eta } (\mathbf{k},\epsilon +i0^{+}))\Big )^{2}\frac{dn_{F}(\epsilon )}{d\epsilon } \end{aligned}$$
